# Supplementary material for: Deletion of wfs1 Impairs Oligodendrocyte Precursor Cells Dorsal Distribution and Myelination Through the wfs1-hmgcs1 Axis in Zebrafish
Source: Biology (Basel). 2026 Aug 16;15(16):1402. doi: 10.3390/biology15161402 (PMC13510151; doi:10.3390/biology15161402)
Supplement: Supplementary file 1 [file biology-15-01402-s001.zip › File S1-Transcriptome sequencing data and differentially expressed genes (DEGs) in wfs1-- zebrafish at 5 dpf/2.KEGG/wfs1KOvsWT/GSEA/wfs1KO/SPLICEOSOME/SPLICEOSOME(DRE03040).html]

Details for gene set SPLICEOSOME(DRE03040)[GSEA]

|  || Dataset | deg.sample |
| Phenotype | sample.cls |
| Upregulated in class | wfs1KO |
| GeneSet | SPLICEOSOME(DRE03040) |
| Enrichment Score (ES) | 0.22145683 |
| Normalized Enrichment Score (NES) | 1.2441819 |
| Nominal p-value | 0.108481266 |
| FDR q-value | 0.32598773 |
| FWER p-Value | 0.981 |
Table: GSEA Results Summary

  

Fig 1: Enrichment plot: SPLICEOSOME(DRE03040)      
 Profile of the Running ES Score & Positions of GeneSet Members on the Rank Ordered List

  

| PROBE | DESCRIPTION (from dataset) | GENE SYMBOL | GENE\_TITLE | RANK IN GENE LIST | RANK METRIC SCORE | RUNNING ES | CORE ENRICHMENT || 1 | 100004402\_1 | plrg1 |  |  | 190 | 4.251 | 0.0449 | Yes |
| 2 | 100037380\_3 | zgc:163098 |  |  | 208 | 4.148 | 0.0940 | Yes |
| 3 | 566017\_1 | lsm8 |  |  | 258 | 3.848 | 0.1385 | Yes |
| 4 | 334283\_2 | ddx23 |  |  | 560 | 2.828 | 0.1629 | Yes |
| 5 | 100005893 | magoh |  |  | 612 | 2.749 | 0.1942 | Yes |
| 6 | 573376 | hspa8 |  |  | 894 | 2.238 | 0.2121 | Yes |
| 7 | 393586 | hsc70 |  |  | 1533 | 1.636 | 0.2116 | Yes |
| 8 | 406264 | hnrpkl |  |  | 2056 | 1.345 | 0.2113 | Yes |
| 9 | 568088 | rbm25a |  |  | 2746 | 1.097 | 0.2027 | Yes |
| 10 | 393282 | snu13a |  |  | 2923 | 1.056 | 0.2098 | Yes |
| 11 | 562935 | si:dkey-4p15.3 |  |  | 2952 | 1.049 | 0.2215 | Yes |
| 12 | 606595 | dhx8 |  |  | 3507 | 0.928 | 0.2151 | No |
| 13 | 394058 | fus |  |  | 3959 | 0.837 | 0.2109 | No |
| 14 | 406288 | srsf1a |  |  | 4232 | 0.788 | 0.2118 | No |
| 15 | 799690\_1 | snrnp200 |  |  | 4645 | 0.728 | 0.2075 | No |
| 16 | 323700 | srsf2b |  |  | 5386 | 0.640 | 0.1919 | No |
| 17 | 286774 | phf5a |  |  | 6159 | 0.572 | 0.1744 | No |
| 18 | 403072 | prpf3 |  |  | 6201 | 0.568 | 0.1799 | No |
| 19 | 550244 | snrpd2 |  |  | 6874 | 0.502 | 0.1647 | No |
| 20 | 798846 | si:ch211-199o1.2 |  |  | 7036 | 0.489 | 0.1655 | No |
| 21 | 57924 | smx5 |  |  | 7220 | 0.472 | 0.1654 | No |
| 22 | 387600 | xab2 |  |  | 7373 | 0.462 | 0.1661 | No |
| 23 | 326944\_2 | prpf4 |  |  | 7661 | 0.442 | 0.1623 | No |
| 24 | 57924\_2 | smx5 |  |  | 8128 | 0.417 | 0.1526 | No |
| 25 | 324121 | snrpa |  |  | 8300 | 0.413 | 0.1522 | No |
| 26 | 393968 | zgc:55733 |  |  | 8725 | 0.386 | 0.1434 | No |
| 27 | 334283 | ddx23 |  |  | 8907 | 0.374 | 0.1422 | No |
| 28 | 406495 | rbm22 |  |  | 8965 | 0.370 | 0.1448 | No |
| 29 | 321544 | prpf19 |  |  | 9532 | 0.331 | 0.1310 | No |
| 30 | 793027\_1 | ccdc12 |  |  | 9622 | 0.325 | 0.1321 | No |
| 31 | 415140 | cwc15 |  |  | 9693 | 0.321 | 0.1337 | No |
| 32 | 446117 | sf3b1 |  |  | 9740 | 0.319 | 0.1361 | No |
| 33 | 394018 | sf3a2 |  |  | 9911 | 0.309 | 0.1344 | No |
| 34 | 402896 | snrpb2 |  |  | 9999 | 0.303 | 0.1353 | No |
| 35 | 415171\_1 | snrpd3 |  |  | 10179 | 0.292 | 0.1332 | No |
| 36 | 334490 | hnrnpub |  |  | 10234 | 0.289 | 0.1349 | No |
| 37 | 492511 | snrpa1 |  |  | 10365 | 0.281 | 0.1342 | No |
| 38 | 572211 | srsf7b |  |  | 10907 | 0.249 | 0.1201 | No |
| 39 | 393436 | aqr |  |  | 10924 | 0.248 | 0.1226 | No |
| 40 | 323071 | tcerg1a |  |  | 11026 | 0.243 | 0.1223 | No |
| 41 | 336645 | dhx16 |  |  | 11674 | 0.207 | 0.1044 | No |
| 42 | 335396 | srsf5a |  |  | 11702 | 0.206 | 0.1060 | No |
| 43 | 494133 | srsf6b |  |  | 11814 | 0.200 | 0.1049 | No |
| 44 | 192325 | ncbp2 |  |  | 11875 | 0.197 | 0.1054 | No |
| 45 | 393951 | prpf8 |  |  | 11890 | 0.196 | 0.1073 | No |
| 46 | 751729 | hnrnpa3 |  |  | 12053 | 0.188 | 0.1044 | No |
| 47 | 393917 | ddx39b |  |  | 12078 | 0.186 | 0.1059 | No |
| 48 | 321931 | dhx15 |  |  | 12349 | 0.171 | 0.0994 | No |
| 49 | 566326 | hnrnpua |  |  | 12589 | 0.160 | 0.0938 | No |
| 50 | 322206 | ddx5 |  |  | 12591 | 0.160 | 0.0957 | No |
| 51 | 192318 | sf3b4 |  |  | 13073 | 0.136 | 0.0822 | No |
| 52 | 406775 | srsf6a |  |  | 13100 | 0.135 | 0.0830 | No |
| 53 | 406352\_1 | hnrnpc |  |  | 13208 | 0.130 | 0.0811 | No |
| 54 | 393920 | crnkl1 |  |  | 13344 | 0.123 | 0.0784 | No |
| 55 | 402989 | u2af2b |  |  | 13420 | 0.120 | 0.0774 | No |
| 56 | 393476 | prpf31 |  |  | 13574 | 0.112 | 0.0740 | No |
| 57 | 406691 | srsf2a |  |  | 13799 | 0.102 | 0.0681 | No |
| 58 | 790924 | usp39 |  |  | 13822 | 0.101 | 0.0686 | No |
| 59 | 393388 | tra2a |  |  | 13928 | 0.096 | 0.0665 | No |
| 60 | 436883 | srsf5b |  |  | 14009 | 0.092 | 0.0650 | No |
| 61 | 406824 | sf3b3 |  |  | 14019 | 0.091 | 0.0659 | No |
| 62 | 378453 | hnrnpa1b |  |  | 14020 | 0.091 | 0.0669 | No |
| 63 | 327495 | acin1b |  |  | 14203 | 0.082 | 0.0622 | No |
| 64 | 368925 | srsf3a |  |  | 14313 | 0.077 | 0.0597 | No |
| 65 | 393480 | eftud2 |  |  | 14533 | 0.068 | 0.0536 | No |
| 66 | 192330 | snrpc |  |  | 14604 | 0.065 | 0.0522 | No |
| 67 | 557103 | u2af2a |  |  | 14701 | 0.060 | 0.0499 | No |
| 68 | 570994 | sf3b2 |  |  | 14854 | 0.053 | 0.0457 | No |
| 69 | 796388 | sf3a1 |  |  | 15194 | 0.037 | 0.0355 | No |
| 70 | 406810 | prpf38b |  |  | 15251 | 0.035 | 0.0341 | No |
| 71 | 321555 | prpf40a |  |  | 15471 | 0.024 | 0.0275 | No |
| 72 | 415211 | puf60b |  |  | 15577 | 0.018 | 0.0244 | No |
| 73 | 326944 | prpf4 |  |  | 15834 | 0.005 | 0.0164 | No |
| 74 | 334283\_1 | ddx23 |  |  | 15949 | 0.000 | 0.0128 | No |
| 75 | 445089 | prpf38a |  |  | 15999 | -0.002 | 0.0113 | No |
| 76 | 368893\_1 | acin1a |  |  | 16031 | -0.002 | 0.0103 | No |
| 77 | 321114 | snu13b |  |  | 16276 | -0.010 | 0.0028 | No |
| 78 | 393889 | rbm17 |  |  | 16505 | -0.017 | -0.0042 | No |
| 79 | 415171 | snrpd3 |  |  | 16565 | -0.019 | -0.0058 | No |
| 80 | 567461 | u2surp |  |  | 16606 | -0.020 | -0.0069 | No |
| 81 | 100037380\_1 | zgc:163098 |  |  | 16745 | -0.024 | -0.0109 | No |
| 82 | 415157 | sf3b6 |  |  | 16840 | -0.028 | -0.0135 | No |
| 83 | 321872 | srsf4 |  |  | 17112 | -0.037 | -0.0216 | No |
| 84 | 558760 | taf15 |  |  | 17315 | -0.046 | -0.0275 | No |
| 85 | 797473 | tcerg1b |  |  | 17322 | -0.046 | -0.0271 | No |
| 86 | 406267 | hnrnpk |  |  | 17435 | -0.051 | -0.0300 | No |
| 87 | 394172 | tra2b |  |  | 17577 | -0.057 | -0.0338 | No |
| 88 | 431751\_1 | prpf18 |  |  | 17727 | -0.064 | -0.0377 | No |
| 89 | 503932 | ddx42 |  |  | 17732 | -0.064 | -0.0371 | No |
| 90 | 493614 | bcas2 |  |  | 17792 | -0.067 | -0.0381 | No |
| 91 | 494165 | ppih |  |  | 17855 | -0.070 | -0.0392 | No |
| 92 | 562370 | puf60a |  |  | 17878 | -0.071 | -0.0391 | No |
| 93 | 321948 | ddx46 |  |  | 17957 | -0.074 | -0.0407 | No |
| 94 | 445302 | thoc3 |  |  | 18025 | -0.077 | -0.0419 | No |
| 95 | 100535720 | lsm3 |  |  | 18026 | -0.077 | -0.0409 | No |
| 96 | 394053 | eif4a3 |  |  | 18040 | -0.078 | -0.0404 | No |
| 97 | 570205 | isy1 |  |  | 18165 | -0.084 | -0.0433 | No |
| 98 | 337851 | cherp |  |  | 18278 | -0.089 | -0.0458 | No |
| 99 | 402966 | snrpb |  |  | 18360 | -0.092 | -0.0472 | No |
| 100 | 327336 | rbm25b |  |  | 18366 | -0.092 | -0.0463 | No |
| 101 | 393840 | ctnnbl1 |  |  | 18385 | -0.093 | -0.0458 | No |
| 102 | 558042 | ppil1 |  |  | 18440 | -0.096 | -0.0463 | No |
| 103 | 323855 | prpf6 |  |  | 18451 | -0.096 | -0.0455 | No |
| 104 | 494534 | cdc40 |  |  | 18756 | -0.111 | -0.0537 | No |
| 105 | 445404\_1 | snrpf |  |  | 18830 | -0.115 | -0.0546 | No |
| 106 | 436976 | snrnp27 |  |  | 18947 | -0.120 | -0.0569 | No |
| 107 | 447922 | snrpg |  |  | 19015 | -0.122 | -0.0575 | No |
| 108 | 449780 | txnl4a |  |  | 19114 | -0.127 | -0.0591 | No |
| 109 | 436751 | sf3b5 |  |  | 19619 | -0.149 | -0.0732 | No |
| 110 | 327011 | snrpd3l |  |  | 20192 | -0.174 | -0.0891 | No |
| 111 | 555643 | hnrnpm |  |  | 20210 | -0.175 | -0.0876 | No |
| 112 | 415167 | lsm6 |  |  | 20243 | -0.177 | -0.0865 | No |
| 113 | 436946 | sart1 |  |  | 20314 | -0.180 | -0.0865 | No |
| 114 | 446168 | sf3a3 |  |  | 20330 | -0.180 | -0.0848 | No |
| 115 | 394059 | cdc5l |  |  | 20386 | -0.182 | -0.0844 | No |
| 116 | 445383 | bud31 |  |  | 20395 | -0.183 | -0.0825 | No |
| 117 | 321860 | rbmx |  |  | 20497 | -0.188 | -0.0834 | No |
| 118 | 393850 | dhx38 |  |  | 20500 | -0.188 | -0.0812 | No |
| 119 | 445043 | syf2 |  |  | 20625 | -0.195 | -0.0828 | No |
| 120 | 192331 | snrpd1 |  |  | 20703 | -0.199 | -0.0828 | No |
| 121 | 572679\_1 | smndc1 |  |  | 20964 | -0.214 | -0.0885 | No |
| 122 | 563948 | lsm5 |  |  | 21277 | -0.229 | -0.0956 | No |
| 123 | 445398 | snrnp70 |  |  | 21372 | -0.235 | -0.0957 | No |
| 124 | 368893 | acin1a |  |  | 21660 | -0.249 | -0.1018 | No |
| 125 | 57924\_1 | smx5 |  |  | 21899 | -0.262 | -0.1061 | No |
| 126 | 560127 | alyref |  |  | 22014 | -0.268 | -0.1065 | No |
| 127 | 192328 | u2af1 |  |  | 22161 | -0.276 | -0.1078 | No |
| 128 | 393979 | snrpe |  |  | 22260 | -0.282 | -0.1075 | No |
| 129 | 393565 | srsf1b |  |  | 22288 | -0.283 | -0.1050 | No |
| 130 | 373077 | thoc1 |  |  | 23005 | -0.328 | -0.1236 | No |
| 131 | 393505 | srsf10b |  |  | 23083 | -0.333 | -0.1221 | No |
| 132 | 405835 | srsf9 |  |  | 23118 | -0.335 | -0.1191 | No |
| 133 | 368417 | thoc2 |  |  | 23234 | -0.343 | -0.1187 | No |
| 134 | 550373 | ppie |  |  | 23552 | -0.364 | -0.1243 | No |
| 135 | 393467 | hnrnpa1a |  |  | 24376 | -0.416 | -0.1453 | No |
| 136 | 503767 | rbm8a |  |  | 24495 | -0.419 | -0.1440 | No |
| 137 | 445404 | snrpf |  |  | 24553 | -0.420 | -0.1407 | No |
| 138 | 393292 | snrnp40 |  |  | 24582 | -0.420 | -0.1366 | No |
| 139 | 57924\_3 | smx5 |  |  | 24690 | -0.429 | -0.1348 | No |
| 140 | 492495 | slu7 |  |  | 24826 | -0.440 | -0.1338 | No |
| 141 | 30671 | hsp70.3 |  |  | 24984 | -0.444 | -0.1334 | No |
| 142 | 321852 | snw1 |  |  | 25021 | -0.447 | -0.1292 | No |
| 143 | 572679 | smndc1 |  |  | 25200 | -0.459 | -0.1293 | No |
| 144 | 573376\_1 | hspa8 |  |  | 25212 | -0.461 | -0.1241 | No |
| 145 | 324476 | zmat2 |  |  | 25344 | -0.470 | -0.1226 | No |
| 146 | 326944\_1 | prpf4 |  |  | 25357 | -0.472 | -0.1174 | No |
| 147 | 100126123 | hsp70.1 |  |  | 25714 | -0.503 | -0.1226 | No |
| 148 | 796523 | pqbp1 |  |  | 26282 | -0.555 | -0.1338 | No |
| 149 | 393669 | lsm4 |  |  | 26327 | -0.559 | -0.1285 | No |
| 150 | 402972 | srsf7a |  |  | 26540 | -0.583 | -0.1282 | No |
| 151 | 492481 | rp9 |  |  | 26718 | -0.600 | -0.1266 | No |
| 152 | 431751 | prpf18 |  |  | 26812 | -0.610 | -0.1222 | No |
| 153 | 573047 | lsm7 |  |  | 28206 | -0.773 | -0.1569 | No |
| 154 | 403072\_1 | prpf3 |  |  | 28850 | -0.886 | -0.1665 | No |
| 155 | 550373\_1 | ppie |  |  | 29974 | -1.233 | -0.1872 | No |
| 156 | 445089\_1 | prpf38a |  |  | 30053 | -1.267 | -0.1745 | No |
| 157 | 406288\_1 | srsf1a |  |  | 30437 | -1.468 | -0.1690 | No |
| 158 | 286774\_1 | phf5a |  |  | 30859 | -1.817 | -0.1605 | No |
| 159 | 394058\_1 | fus |  |  | 31078 | -2.053 | -0.1428 | No |
| 160 | 100005893\_1 | magoh |  |  | 31134 | -2.131 | -0.1190 | No |
| 161 | 566017 | lsm8 |  |  | 31384 | -2.610 | -0.0956 | No |
| 162 | 560210 | hsp70l |  |  | 31396 | -2.640 | -0.0643 | No |
| 163 | 100004402 | plrg1 |  |  | 31417 | -2.696 | -0.0327 | No |
| 164 | 100037380\_2 | zgc:163098 |  |  | 31736 | -3.974 | 0.0049 | No |
Table: GSEA details [plain text format]

  

Fig 2: SPLICEOSOME(DRE03040)      
 Blue-Pink O' Gram in the Space of the Analyzed GeneSet

  

Fig 3: SPLICEOSOME(DRE03040): Random ES distribution      
 Gene set null distribution of ES for **SPLICEOSOME(DRE03040)**
